# Assessing the ABL 500 blood gas analyser

**DOI:** 10.1155/S1463924691000196

**Published:** 1991

**Authors:** B. Gouget, R. Andriamahatratra, Y. Gourmelin, A. Truchaud

**Affiliations:** Laboratoire de Biochimie Centre hospitalier général, BP 218 Meaux F77108 France

## Abstract

The ABL 500 blood gas analyser from Radiometer has cordless electrodes and does not use a humidifier for calibrating gases. During the evaluation of the analytical performance of this instrument, the problem of p0_2_ accuracy was approached by comparing the values obtained with two kinds of tonometry (film and bubble). An acceptable level of imprecision was demonstrated for all measured parameters. For within-run precision, with tonometry, coefficients of variation (CV) were ≆0.37% for pO_2_ and ≆0.52% for pCO_2_. A CV of 1.76% was found for day-to-day precision for both p0_2_ and pCO_2_. In the linearity study, with both tonometry methods, and in the inter-instrument comparisons (the ABL was compared with the Ciba Corning 178), pO_2_ values obtained on the ABL 500 exhibited a slight overestimation above 150 mmHg (2.2-3.4% at 600 mmHg). This minor discrepancy is discussed with reference to the new design of the pO_2_ electrode, the algorithm for pO_2_ correction and the tonometry procedure. The results reported in this paper stress the importance of pO_2_ accuracy assessment for the evaluation of blood gas analysers.


Journal of Automatic Chemistry, Vol. 13, No. 3 (May–June 1991), pp. 101–106

### Assessing the ABL 500 blood gas analyser

**B. Gouget, R. Andriamahatratra, Y. Gourmelin and A. Truchaud**

*Laboratoire de Biochimie, Centre hospitalier général, BP 218, F77108 Meaux, France*

*The ABL 500 blood gas analyser from Radiometer has cordless electrodes and does not use a humidifier for calibrating gases. During the evaluation of the analytical performance of this instrument, the problem of $pO_2$ accuracy was approached by comparing the values obtained with two kinds of tonometry (film and bubble). An acceptable level of imprecision was demonstrated for all measured parameters. For within-run precision, with tonometry, coefficients of variation (CV) were $\leqslant$0·37% for $pO_2$ and $\leqslant$0·52% for $pCO_2$. A CV of 1·76% was found for day-to-day precision for both $pO_2$ and $pCO_2$. In the linearity study, with both tonometry methods, and in the inter-instrument comparisons (the ABL was compared with the Ciba Corning 178), $pO_2$ values obtained on the ABL 500 exhibited a slight overestimation above 150 mmHg (2·2–3·4% at 600 mmHg). This minor discrepancy is discussed with reference to the new design of the $pO_2$ electrode, the algorithm for $pO_2$ correction and the tonometry procedure. The results reported in this paper stress the importance of $pO_2$ accuracy assessment for the evaluation of blood gas analysers.*

The Radiometer ABL 500 blood gas analyser is modular and includes some highly innovative features, such as cordless electrodes and a remembraning system. The analyser was evaluated for analytical performance and practicability, using both tonometry and commercial aqueous control solutions [1]. A recurrent problem in evaluation in blood gas instrumentation is $pO_2$ accuracy [2] – built-in corrections apply to the directly measured $pO_2$ values. The accuracy of $pO_2$ displayed by the instrument depends upon the quality of the algorithms used for correction.

The equation needed for this adjustment is determined empirically during the development of a new instrument by comparing data from several uncorrected analysers to a reference method [3]. There is no standard method for determining the partial pressures of blood gases with absolute accuracy [4]. However, tonometry of fresh whole blood is considered the reference method for assessing $pO_2$ accuracy. In order to test the validity of the ABL 500 algorithm for $pO_2$ correction, two kinds of tonometry were used (film and bubble) and the reliability of $pO_2$ measurement was assessed according to the results obtained by the two methods.

### Materials and methods

*Blood gas analysers*

The ABL 500 measures pH, $pCO_2$ and $pO_2$. Electrodes are based upon reference technology with miniaturized cordless electrodes, which are colour coded to ensure correct placement. The built-in gas mixer supplies the following gas mixtures from atmospheric air and from pure $CO_2$: G1: 5·6% $CO_2$, 19·76% $O_2$; G2: 10% $CO_2$ is the high point of calibration of the $pCO_2$ electrode; G3 is 100% $CO_2$ and is used for the zero calibration of the $pO_2$ electrode. The ever-present film of rinse solution inside the tubing is sufficient to achieve the necessary humidification of the calibration gases. The pH electrode is calibrated using two reagent buffers. Automatic calibrations are adjusted according to individual requirements. The ABL 500 accepts two sample sizes: 35 µl for pH in a special micromode; a total blood gas analysis is available from 70 µl using injection or an aspiration mode.

The Ciba Corning 178 blood gas analyser was used as comparison instrument.

*Materials*

*Solutions*

Phosphate buffer solutions at pH 6·838 and 7·384 were prepared in line with the recommendations of National Bureau of Standards [5].

Qualicheck Radiometer aqueous control solutions were used at three levels (acidosis, normal and alkalosis) and a fourth high oxygen level was used for the within-run study.

*Tonometers*

Tonometry was performed according to the IFCC reference method [6] on a Laue Bulb (Eschweiler, Germany) and on the Corning 184 (Ciba-Corning Medical and Scientific, Medfield, Massachusetts, USA) tonometers.

The Laue bulb tonometer consists of a rotating glass bulb, placed in a water-bath at 37·0 °C with a gas humidifier and a gas vent. Due to the excentric rotation of the bulb a thin film of the sample is formed. The Corning 184 tonometer is designed as a syringe tonometer according to the bubble equilibration principle. Antifoam solution was added to blood samples (Corning antifoam solution). Both tonometers were operated and maintained according to the manufacturer's instruments.

*Blood samples*

Fresh heparinized venous or arterial blood was obtained from healthy donors for tonometry and from hospitalized patients for comparison studies.

### Protocol

*pH accuracy*

The two phosphate buffers were run in triplicate over five days.

0142–0453/91 $3.00 © 1991 Taylor & Francis Ltd.

*Table 1. Within-run precision ($N = 6$).*

| Qualicheck solutions | | | | | | | | | |
|---|---|---|---|---|---|---|---|---|---|
| | | Mean | ±SD | Mean | ±SD | Mean | ±SD | Mean | ±SD |
| pH | | 7·121 | 0·002 | 7·385 | 0·001 | 7·610 | 0·001 | 7·108 | 0·002 |
| | | Mean | CV% | Mean | CV% | Mean | CV% | Mean | CV% |
| $pCO_2$ | mmHg | 59·1 | 0·26 | 39·8 | 0·43 | 18·2 | 0·33 | 102·4 | 0·43 |
| | (kPa) | (7·89) | | (5·31) | | (2·43) | | (13·65) | |
| $pO_2$ | mmHg | 54·9 | 0·73 | 106·2 | 0·29 | 171·8 | 0·27 | 305·1 | 0·47 |
| | (kPa) | (7·32) | | (14·15) | | (22·90) | | (40·67) | |

| Blood tonometry | | | | | | | |
|---|---|---|---|---|---|---|---|
| | | Assigned value | Mean | CV% | Assigned value | Mean | CV% |
| $pCO_2$ | mmHg | 20 | 20·3 | 0·36 | 80 | 81·3 | 0·52 |
| | (kPa) | (2·66) | (2·70) | | (10·64) | (10·84) | |
| $pCO_2$ | mmHg | 40 | 40·1 | 0·37 | 160 | 158·8 | 0·30 |
| | (kPa) | (5·32) | (5·33) | | (21·28) | (21·17) | |

*Precision study*

*Within-run precision* was estimated with Qualicheck solutions and two levels of tonometry (L1: $CO_2$ = 20 mmHg [2·66 kPa], $O_2$ = 40 mmHg [5·32 kPa]; L2: $CO_2$ = 80 mmHg [10·64 kPa] $O_2$ = 160 mmHg [21·28 kPa]). Six measurements were performed at each level.

*Day-to-day precision*

The same solutions and same levels of tonometry were run daily for 25 days.

Drift was assessed using tonometered blood tested immediately after one calibration and before the subsequent one.

Linearity was tested using successive measurements of tonometered blood containing $O_2$ for 0 to 85% and $CO_2$ from 1 to 20%. The sequence was repeated three times with the two systems of tonometry.

*Inter-instrument comparisons*

About 300 samples from patients were simultaneously measured on both instruments. The values covered the patho-physiological ranges for the three parameters under investigation.

*Practicability*

Special attention was paid to quantifying the most important specifications of the instrument, to maintenance requirements and to safety.

### Results

The mean pH values from the triplicate measurements were never separated by ±0·01 UpH from the assigned values of the two buffers.

*Precision study*

*Within run precision*

Coefficients of variation never exceeded 0·43% for $pCO_2$ and 0·73% for $pO_2$ at each level of Qualicheck solutions. With film tonometry CVs were ≤0·52% for $pCO_2$ and ≤0·37% for $pO_2$. Results for a representative sequence of the within run precision study are given in table 1.

Results for *day-to-day precision* are given in table 2 and illustrate that the measured values by tonometry were very close to the assigned values.

*Linearity*

For $pCO_2$, linearity was verified between 0 and 150 mmHg (0 and 20 kPa). Figure 1 reports the aggregate results for $pO_2$ linearity by film tonometry between 0 and 600 mmHg (80 kPa) with a simple regression line $y = 1{\cdot}03x–1{\cdot}09$; $r > 0{\cdot}999$. When the data were examined in more detail, two patterns of linearity could be observed (see figure 2). In the range 0–150 mmHg (20 kPa), the assigned and measured values were identical. Above 150 mmHg (20 kPa), a slight over-estimation was observed (+2·2% at 600 mmHg (80 kPa)); using bubble tonometry, this discrepancy was even larger (+3·4% at 600 mmHg (80 kPa)) (see figure 3). Differences between $pO_2$ results obtained under aspiration and injection modes, with film tonometry, were found to be statistically significant above 350 mmHg (8·2 and 10·4 mmHg at 500 and 600 mmHg respectively), but not clinically unacceptable.

No drift was observed between calibrations.

*Inter-instrument comparisons*

Figures 4, 5 and 6 report data on the inter-instrument comparisons for pH, $pCO_2$, $pO_2$ and indicate that values for pH (6·95–7·63) were similar on the ABL 500 and Ciba Corning 178 analysers. $pCO_2$ values on the ABL 500 (10–102 mmHg) were slightly lower than on the Ciba Corning 178 (the means were 40·3 and 41·5 mmHg, respectively). This is not unexpected, because $pCO_2$ values on the Ciba

*Table 2. Day-to-day precision (N = 25).*

| Qualicheck solutions | | Mean | ±SD | Mean | ±SD | Mean | ±SD |
|---|---|---|---|---|---|---|---|
| pH | | 7·120 | 0·003 | 7·376 | 0·004 | 7·611 | 0·002 |
| | | Mean | CV% | Mean | CV% | Mean | CV% |
| $pCO_2$ | mmHg | 59·6 | 0·97 | 39·8 | 1·20 | 18·3 | 1·90 |
| | (kPa) | (7·94) | | (5·31) | | (2·45) | |
| $pO_2$ | mmHg | 54·6 | 1·95 | 104·3 | 1·60 | 172·2 | 0·84 |
| | (kPa) | (7·28) | | (13·90) | | (22·96) | |

| Blood tonometry | | Assigned value | Mean | CV% | Assigned value | Mean | CV% |
|---|---|---|---|---|---|---|---|
| $pCO_2$ | mmHg | 20 | 20·5 | 1·76 | 80 | 82·6 | 1·07 |
| | (kPa) | (2·66) | (2·73) | | (10·64) | (11·01) | |
| $pCO_2$ | mmHg | 40 | 40 | 1·75 | 160 | 157·8 | 1·05 |
| | (kPa) | (5·32) | (5·32) | | (21·28) | (21·03) | |

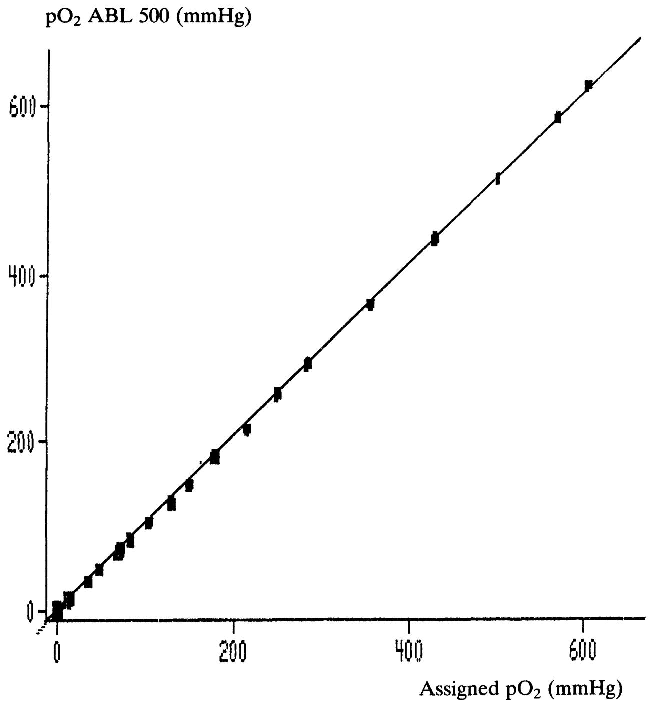

*Figure 1. $pO_2$ linearity on the ABL 500 analyser using film tonometry (N = 134) between 0 and 600 mmHg (y = 1·03, x − 1·09, r ≥ 0·999).*

Corning 178 are often higher than on other modern analysers (this was demonstrated by a recent French interlaboratory quality control program). For $pO_2$ (12–432 mmHg (1·6–57·5 kPa)), a trend for overestimation on the ABL 500 versus Ciba Corning 178 above 150 mmHg (20 kPa) was observed.

*Practicability*

The ABL 500 is based on a modular design with a wet section located in the front part of the measuring station, sensitive electronics in an independent module, and a small $CO_2$ cylinder inside a cabinet frame, saving space and costs. The measuring chamber is clearly visible. Remembraning time is reduced with disposable membrane units, which are prefilled with electrolytes solutions. The PC-style software is ingenious and easily accessible (finger wheel, pop-up windows etc.). Maintenance is guided by an adaptable self-diagnostic program. The mechanical steps are simple and easily learnt. Reagents and waste containers are designed to conform to strict biological safety rules. The inlet section provides easy access for sample introduction and cleaning of the protective flap. The automatic results print-out is easily edited and items can be selected from it.

### Discussion

The evaluation demonstrated a high degree of precision for all measured parameters. Inter-instrument comparisons and reference method application identified some discrepancies especially for high $pO_2$ values.

The linearity study gave the following results:

(1) The zero point calibration was verified by tonometry. The measurement of the electrode response, at zero $pO_2$, increases the accuracy of the subsequent determinations.

(2) Near the second calibration point of the slope ($O_2$ = 20%), the measured values were identical to the expected ones.

(3) Discrepancies appeared above 150 mmHg (20 kPa) of $pO_2$. Several hypotheses can be discussed.

The values given by the analyser are always corrected values. The algorithm for correction is dependent upon the gas/liquid ratio and the sensitivity of the $pO_2$ electrode, calculated during the last calibration of the high $pO_2$ gas. There are also corrections for systematic deviations arising from the contamination of the sample by the amount of oxygen present on the inner walls of the tubing and/or measuring chamber. The magnitude of this effect is influenced by different factors, such as the

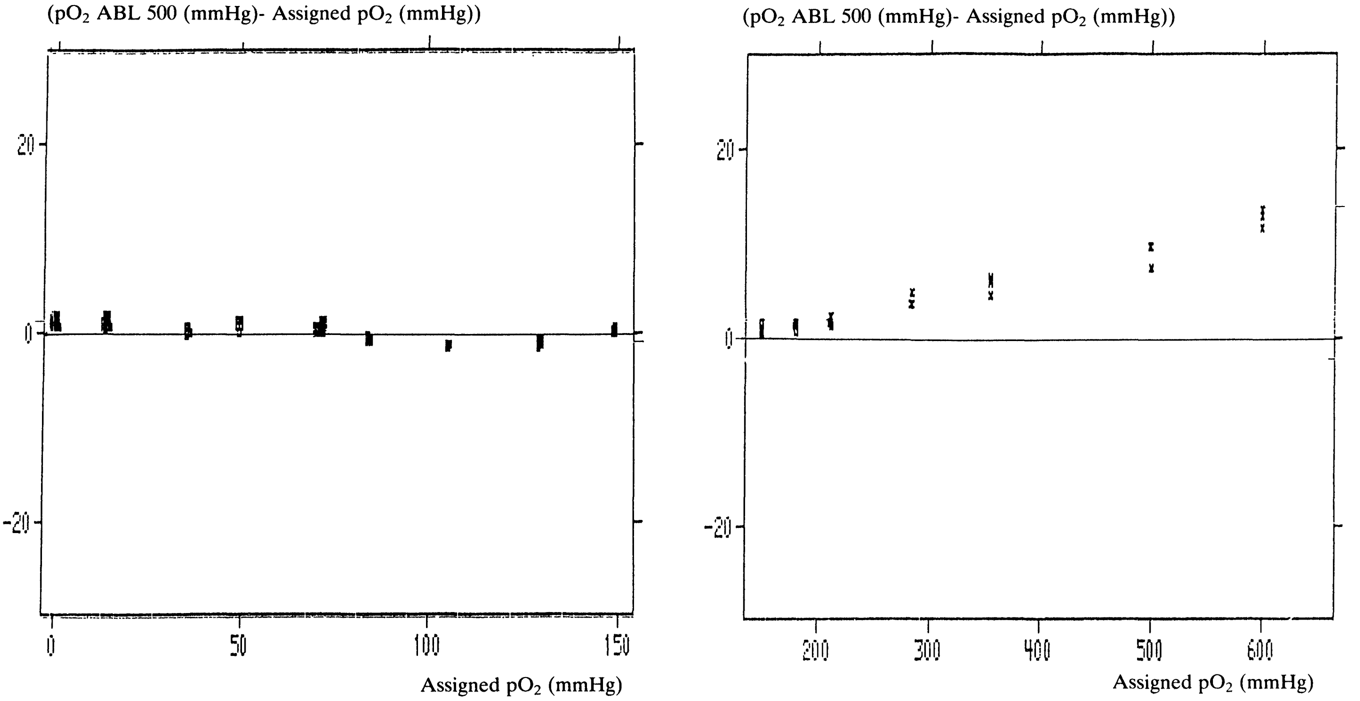

*(a)* *(b)*

*Figure 2. $pO_2$ linearity for low (0–150 mmHg [0–20 kPa], N = 101) (a), and high (150–600 mmHg [20–80 kPa], N = 33] (b), $pO_2$ values with film tonometry. The x axis represents assigned $pCO_2$ values with film tonometry; and the y axis represents the difference between the actual values measured on ABL 500 and the assigned values.*

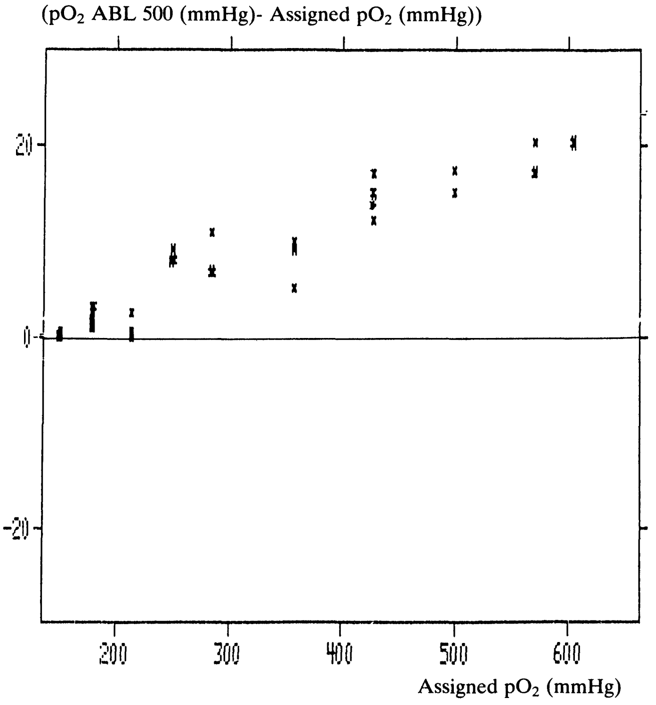

*Figure 3. $pO_2$ linearity using bubble tonometry between 150 and 600 mmHg (20–80 kPa), N = 50. x and y axes as figure 2.*

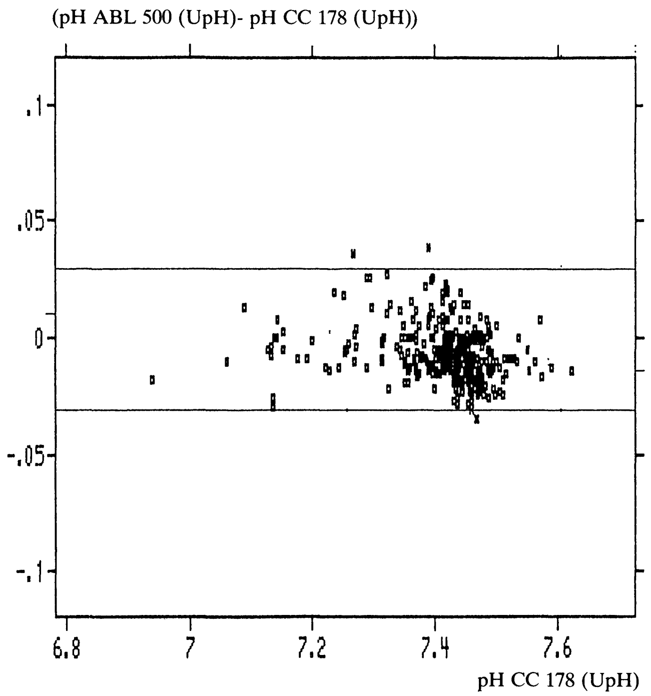

*Figure 4. Inter-instrument comparison for pH, N = 291 samples for hospitalized patients (y = 0·975x +0·18, r = 0·992). The x axis represents measurements performed on Ciba Corning 178 analyser; and the y axis represents the difference in pH values between the ABL 500 and the Ciba-Corning 178 analysers. The solid horizontal lines represent pre-established limits (±0·03 UpH) of acceptable imprecision.*

ratio between sample volume and contact surface, the oxygen buffer capacity of the sample and the surface layer of the walls and finally the time of contact between sample and wall.

Although tonometered, fresh blood remains the best means for testing instrument accuracy. It requires a strict

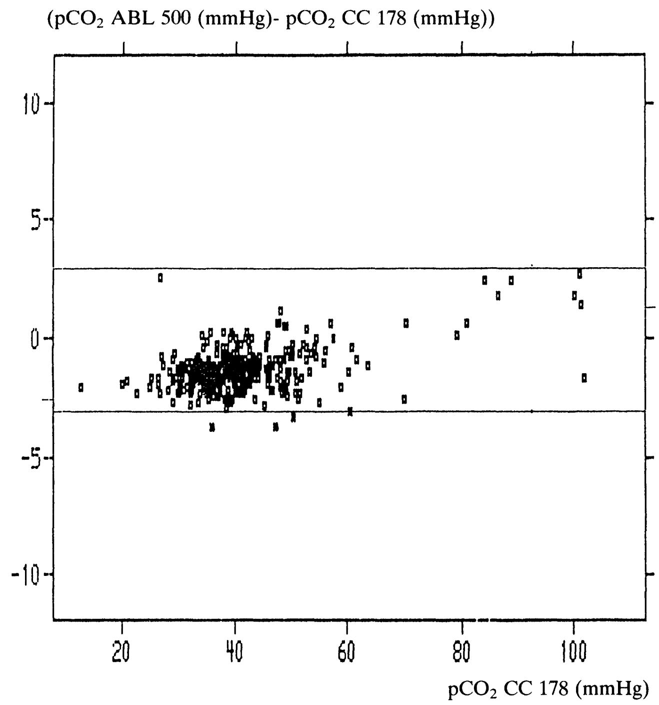

*Figure 5. Inter-instrument comparison for $pCO_2$ (N = 291), y and x axes as figure 4. The pre-established limits for $pCO_2$ are ±3 mmHg. ($y = 1{\cdot}04x - 2{\cdot}71$, $r = 0{\cdot}998$).*

control of working conditions: the IFCC method was followed for tonometry of blood, particularly in terms of procedure and equilibrating conditions [6]. Improper handling during the anaerobic use of the Laue tonometer and gas humidification, for instance, yield inaccurate values [7]. Even at gas flow rates of 60 ml/min and

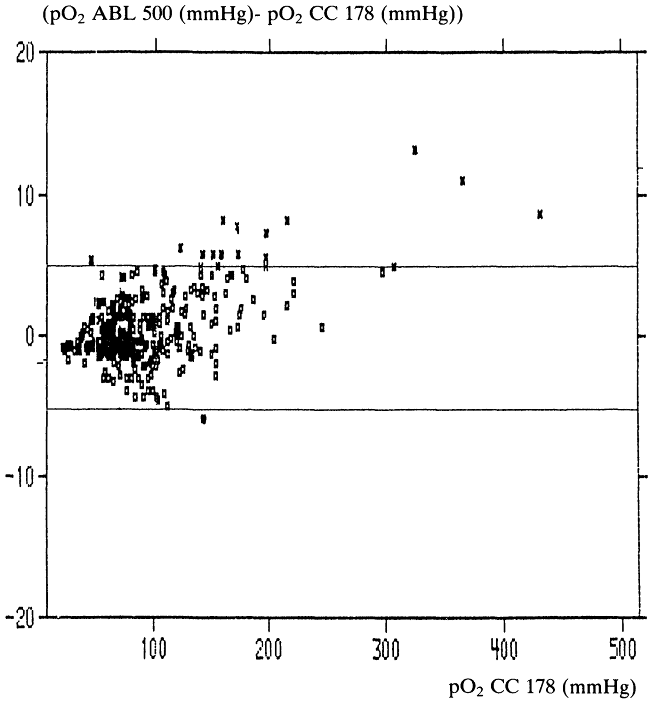

*Figure 6. Inter-instrument comparison for $pO_2$ (N = 291), y and x axes as figure 4. The pre-established limits for $pO_2$ are ±5 mmHg. ($y = 1{\cdot}03x + 1{\cdot}94$, $r = 0{\cdot}999$).*

equilibrating temperature maintained at 37·0 ± 0·10 °C could not account for the differences observed between the assigned and the measured values at high $pO_2$ levels. In the Corning 184 tonometer, the blood sample is transferred directly from the equilibration syringe to the blood gas instrument, but changes in the sample may have several explanations:

(*a*) Haemolysis due to the antifoam solution, but the consequence of this is minor magnitude [8].

(*b*) A bubble effect, which raises $pO_2$. This bubble effect is related to the surface tension of the liquid surrounding the bubble, the bubble diameter and the hydrostatic pressure in the tonometer vessel [9].

The difference in $pO_2$ between the Corning 184 and the Laue tonometer at a level of 600 mmHg (80 kPa) was 7·5 mmHg (1 kPa) and was due to the bubble effect. An estimation of this bubble effect has previously been calculated at 13·5 mmHg (1·8 kPa) for $pO_2$ at 675 mmHg (90 kPa) between the Corning 184 and IL 237 tonometers [10].

However, beside the discrepancies demonstrated by tonometry, an overestimation of high $pO_2$ values persists on the ABL 500 analyser. For example, the measuring time on blood samples is not constant for the $pO_2$ electrode. The value calculated from the second reading is used to determine the measuring time. When $pO_2$ is very low or very high, this time is maximal. In addition, the inner side of the polypropylene membrane is covered by platinum black, resulting in a faster and more stable $pO_2$ electrode. Theoretically, these new features should have improved the stability and the accuracy of the $pO_2$ electrode.

The algorithm was established using capillary tubes and aspiration mode, as on the previous models of analysers by Radiometer [11]. Under these conditions the volume of the sample and the magnitude of the contamination were precisely known. However, for the present evaluation, syringes and the injection mode were used. The volume of the sample is probably larger and more variable and the contamination not identical to the conditions defined by the aspiration mode. However, the injection mode with a syringe is more generally used for routine measurements. Moreover, $pO_2$ values were not identical above 150 mmHg (20 kPa) between the Ciba Corning 178 and the ABL 500, when comparing patients' samples. These two facts suggest that the small overestimations of the ABL 500 $pO_2$ determinations at elevated oxygen pressures are of minor importance. Difficulties in handling samples with very high oxygen tensions must be noted. Most performance specifications given by the manufacturers do not reach $pO_2$ values above 550 mmHg. To further improve $pO_2$ accuracy, the algorithm for correction should take into account high $pO_2$ tensions and the mode of sample introduction.

In conclusion, this study emphasizes that assessment of $pO_2$ accuracy is still difficult. The quality control materials which are available today are not ideal. However, commercially prepared materials are appropriate on a routine basis [12]. Tonometry is still questionable, but it is the only way to assess the characteristic properties of the blood gas analysers: imprecision, inaccuracy and inter-instrument variations [13]. The slight inaccuracy observed for hyperoxic levels on the ABL 500 analyser should be balanced by the reduced clinical interest in the $pO_2$ reliability in this high range. The technical innovations associated with the up-to-date computer style of this analyser makes it particularly easy to run after a short period of training.

### References

1. Gouget, B., Gourmelin, Y., Feuillu, A., Blanchet, F., Capolaghi, B., Lagente, M., Lardet, G., Manceaux, J. P. and Truchaud, A., *Journal of Automatic Chemistry*, **11** (1989), 266–272.
2. Eichhorn, J. H., *Chest*, **94** (1988), 1–2.
3. *ABL 500 Blood gas system user's handbook* (Radiometer, 1989), book 4.
4. Christiansen, T. F., In *Methodology and Physiology of Blood Gas and pH. Proceedings of the 6th Meeting of the IFCC Expert Panel on pH and Blood Gases* (Groningen, 23–25 August, 1981), 67.
5. Durst, R. A., *Standard Reference Materials. Standardization of pH Measurement* (National Bureau of Standards, Washington D.C., 1975).
6. Burnett, R. W., Covington, A. K., Maas, A. H. J., Muller-Plathe, O., Weisberg, H. F., Winberley, P. D., Zijlstra, W. G., Siggaard-Andersen, O. and Durst, R. A., *Journal of Clinical Chemistry and Clinical Biochemistry*, **27** (1989), 403.
7. Farhi, H., *Journal of Applied Physiology*, **20** (1965), 1098–1101.
8. Leary, E. T., Delaney, C. J. and Kenny, D. A., *Clinical Chemistry*, **23** (1977), 493–503.
9. Ravin, M. B. and Briscoe, W. A., *Journal of Applied Physiology*, **19** (1964), 784–790.
10. Sprokholt, R. and Maas, A. H. J., In *Methodology and Physiology of Blood Gases pH. Proceedings of the 6th Meeting of the IFCC Expert Panel on pH and Blood Gases* (Groningen, 23–25 August, 1981), 28–50.
11. Holbeck, C. C., *Journal of Clinical Monitoring*, **5** (1989), 4–16.
12. Hansen, J. E. and Feil, M. J ., *Chest*, **94** (1988), 49–54.
13. Van Kessel, A. L., Eichhorn, J. H., Clausen, J. L., Stone, M. E., Rotman, H. H. and Crapo, R. O., *Chest*, **92** (1987), 418–422.

